# Case Report: Rare Müllerian malformation assisted by digital 3D CT reconstruction technology for diagnosis and treatment

**DOI:** 10.3389/fmed.2025.1657613

**Published:** 2025-11-13

**Authors:** Xiuzhen Wang, Genhai Zhu

**Affiliations:** Department of Gynecology, Hainan General Hospital (Hainan Affiliated Hospital of Hainan Medical University), Haikou, China

**Keywords:** Müllerian malformation, digital 3D CT reconstruction, rudimentary horn uterus, unilateral adnexal absence, primary amenorrhea

## Abstract

**Introduction:**

Menstruation marks a significant developmental milestone, typically indicating healthy and normal pubertal changes in females. Primary amenorrhea can arise from various causes, including outflow tract abnormalities, resistant endometrium, primary ovarian insufficiency, and disorders of the hypothalamus, pituitary gland, or other endocrine organs. This report presents an exceptionally rare Müllerian malformation that does not conform perfectly to existing classification systems, necessitating an individualized management approach.

**Case presentation:**

We present the case of a 12-year-old girl with a rare Müllerian malformation, who presented with cyclic lower abdominal pain persisting for over 9 months, with acute exacerbation in the preceding day. Comprehensive clinical evaluation and preoperative pelvic CT imaging revealed multiple anomalies: an unilateral rudimentary horn uterus with cavity and unilateral absence of the ovary and fallopian tube. Digital 3D CT reconstruction was utilized to confirm the diagnosis, revealing a left rudimentary horn uterus with cavity, hydrosalpinx of the left fallopian tube, and absence of the right ovary, fallopian tube and hemi-uterus. Surgical management included laparoscopic resection of the left rudimentary horn uterus and left fallopian tube. Postoperatively, the patient achieved complete resolution of cyclic lower abdominal pain.

**Conclusion:**

Digital 3D CT reconstruction technology is instrumental in diagnosing rare Müllerian malformations, providing crucial anatomical insights for surgical planning and patient counseling.

## Introduction

Menstruation is a critical developmental milestone in females, typically signifying the onset of healthy and normal pubertal changes. However, disruptions in this process, such as primary amenorrhea, can arise from a variety of etiologies, including outflow tract abnormalities, resistant endometrium, primary ovarian insufficiency, and disorders of the hypothalamus, pituitary gland, or other endocrine organs (1). Among these, Müllerian malformations represent a rare but significant cause of primary amenorrhea and cyclic pelvic pain, often requiring a nuanced and individualized approach to diagnosis and management (2).

Müllerian duct anomalies (MDAs) are congenital abnormalities resulting from incomplete development or fusion of the Müllerian ducts during embryogenesis. These anomalies can manifest in diverse forms, ranging from uterine agenesis to complex structural defects such as unicornuate uterus, didelphys uterus, or rudimentary horn uterus (3). While classification systems, such as the American Society for Reproductive Medicine (ASRM) classification, provide a framework for categorizing these malformations, some complex cases pose challenges for conventional categorization due to their unique anatomical features (4). Such cases necessitate advanced diagnostic tools, such as digital three-dimensional (3D) computed tomography (CT) reconstruction, to accurately delineate the anatomical abnormalities and guide surgical intervention (5).

Here, we present a rare case of Müllerian malformation in a 12-year-old girl with atypical features that do not align with existing classification systems. This case underscores the importance of advanced imaging techniques and a tailored approach to management in addressing complex congenital anomalies of the female reproductive tract.

## Case presentation

A 12-year-old premenarchal girl with no history of sexual activity was admitted on April 13, 2024, presenting with cyclical lower abdominal pain persisting for over 9 months, which had acutely worsened over the preceding day. The patient had never experienced menstruation.

Gynecological examination upon admission: External genitalia: Normal development, no abnormalities observed. Vagina: Intact hymen, vaginal length approximately 8 cm, with a blind-ending vault. Rectal examination: No palpable pelvic masses.

**Figure 1:**
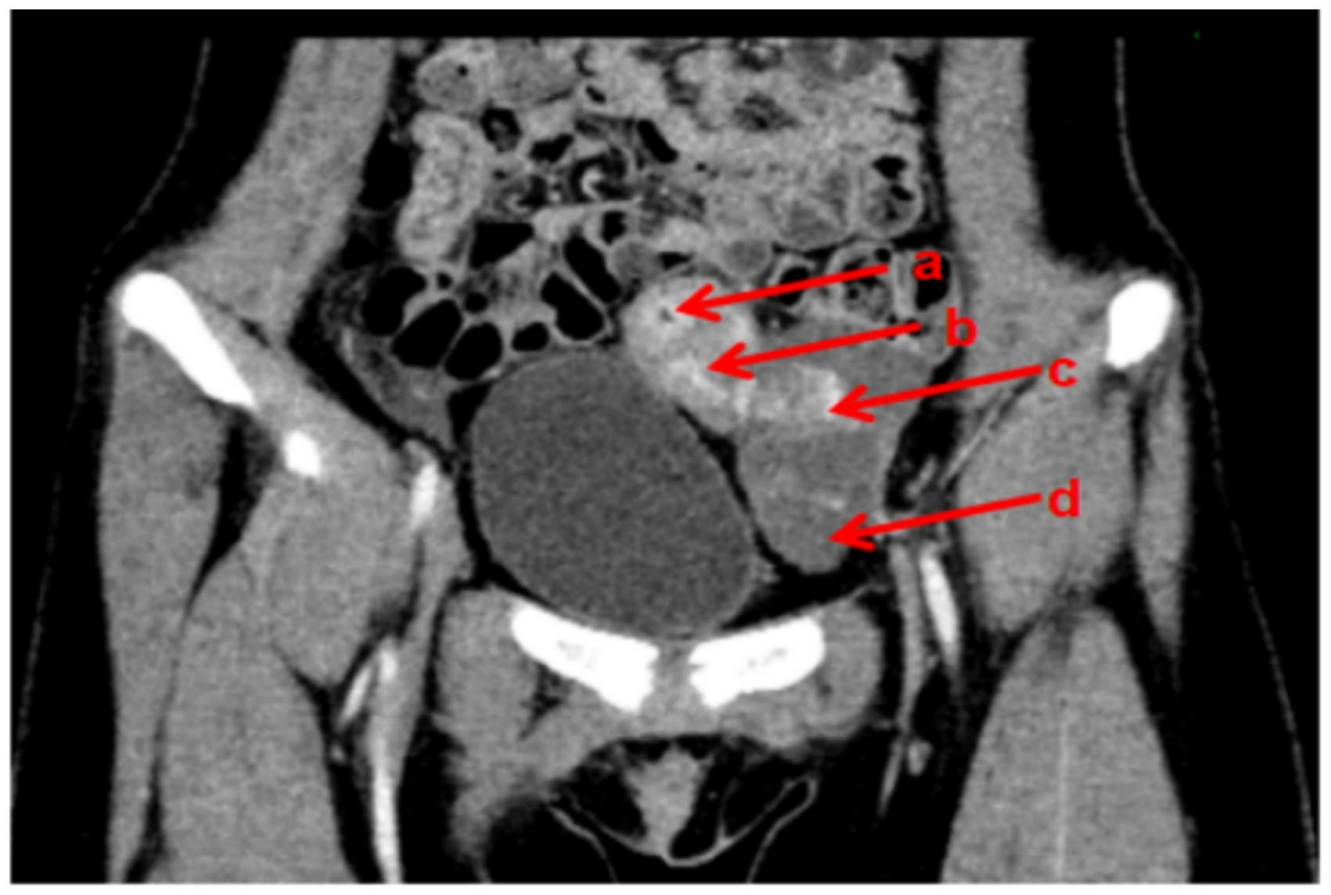
Preoperative CT images of the patient (a: uterine cavity; b: left rudimentary horn uterus; c: left ovary; d: left fallopian tube).

**Figure 2:**
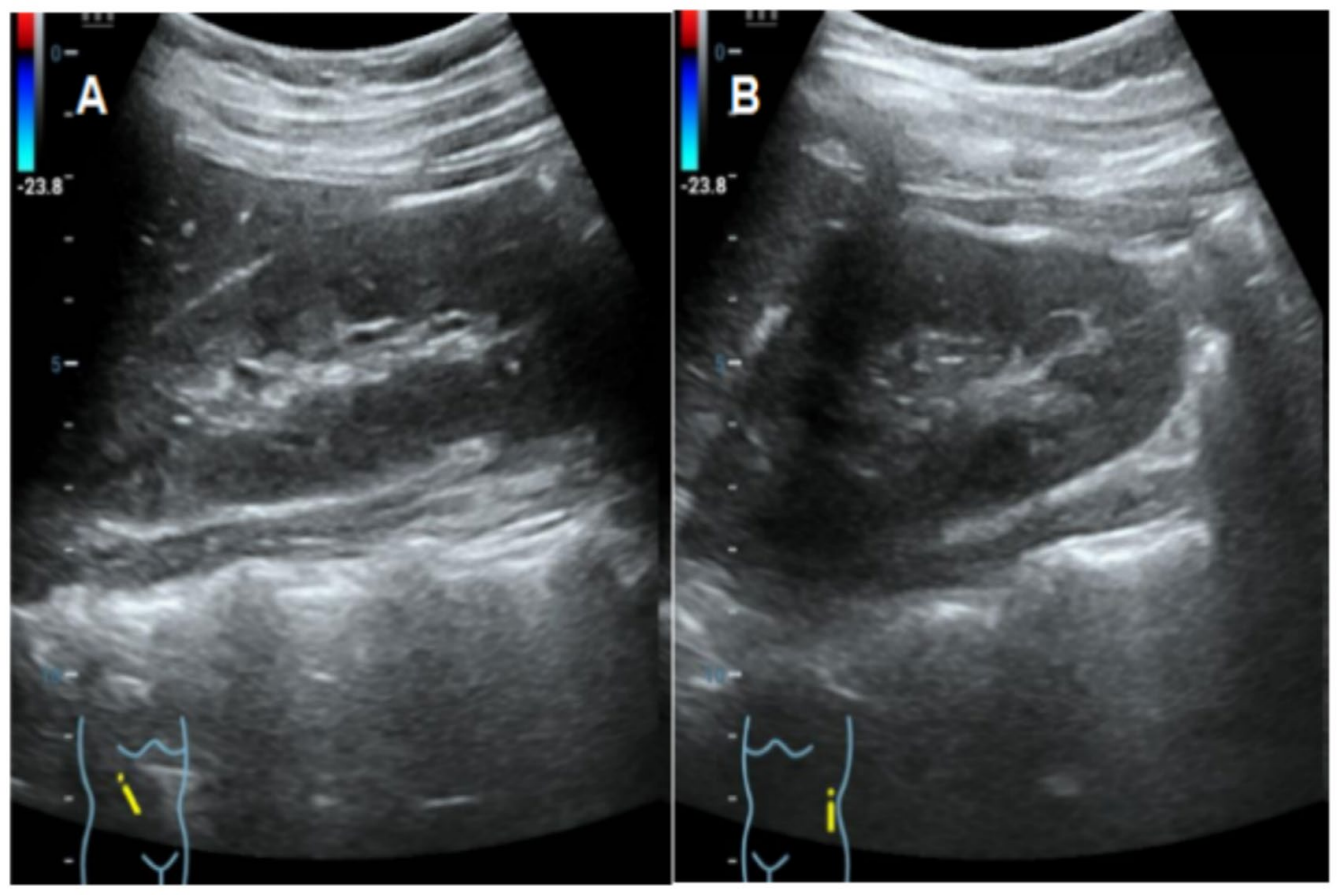
Ultrasound images of the patient’s urinary system (**A**: right kidney; **B**: left kidney).

Auxiliary examinations: (1) Gynecological ultrasound (including both transabdominal and transrectal approaches): Uterus located on the left side of the pelvis, displaying abnormal morphology with no discernible cervical structure. Right ovary not visualized, suggestive of a rudimentary horn uterus. The uterine myometrium exhibited heterogeneous echotexture, suggestive of adenomyosis. Cystic mass in the left adnexal region, consistent with hydrosalpinx of the left fallopian tube. (2) Pelvic Computed Tomography (CT) with Contrast and Plain Scan: Left rudimentary horn uterus, potentially containing an endometrial cavity. Irregular left ovary. Dilated and hydropic left fallopian tube. Right ovary not clearly visualized (Figure 1). (3) Urinary system ultrasound: No abnormalities detected in the kidneys, ureters, or bladder (Figure 2). (4) Chromosome karyotype: 46, XX. (5) Sex Hormone Panel (April 18, 2024): Luteinizing hormone (LH): 1.41 IU/L (↓), Follicle-stimulating hormone (FSH): 5.66 IU/L, Estradiol (E2): 125.0 pmol/L, Progesterone: 0.43 nmol/L, Prolactin: 127.3 mIU/L, Testosterone: 1.05 nmol/L.

The clinical timeline was as follows: Cyclical lower abdominal pain began approximately 9 months prior to admission (around July 2023). The patient presented to our hospital on April 13, 2024, due to an acute exacerbation. Comprehensive diagnostic evaluations were completed between April 13 and April 18, 2024. The therapeutic laparoscopic surgery was performed on April 19, 2024. Postoperative follow-up continued for 9 months (until approximately January 2025), during which the patient reported complete and sustained resolution of her symptoms.

**Figure 3:**
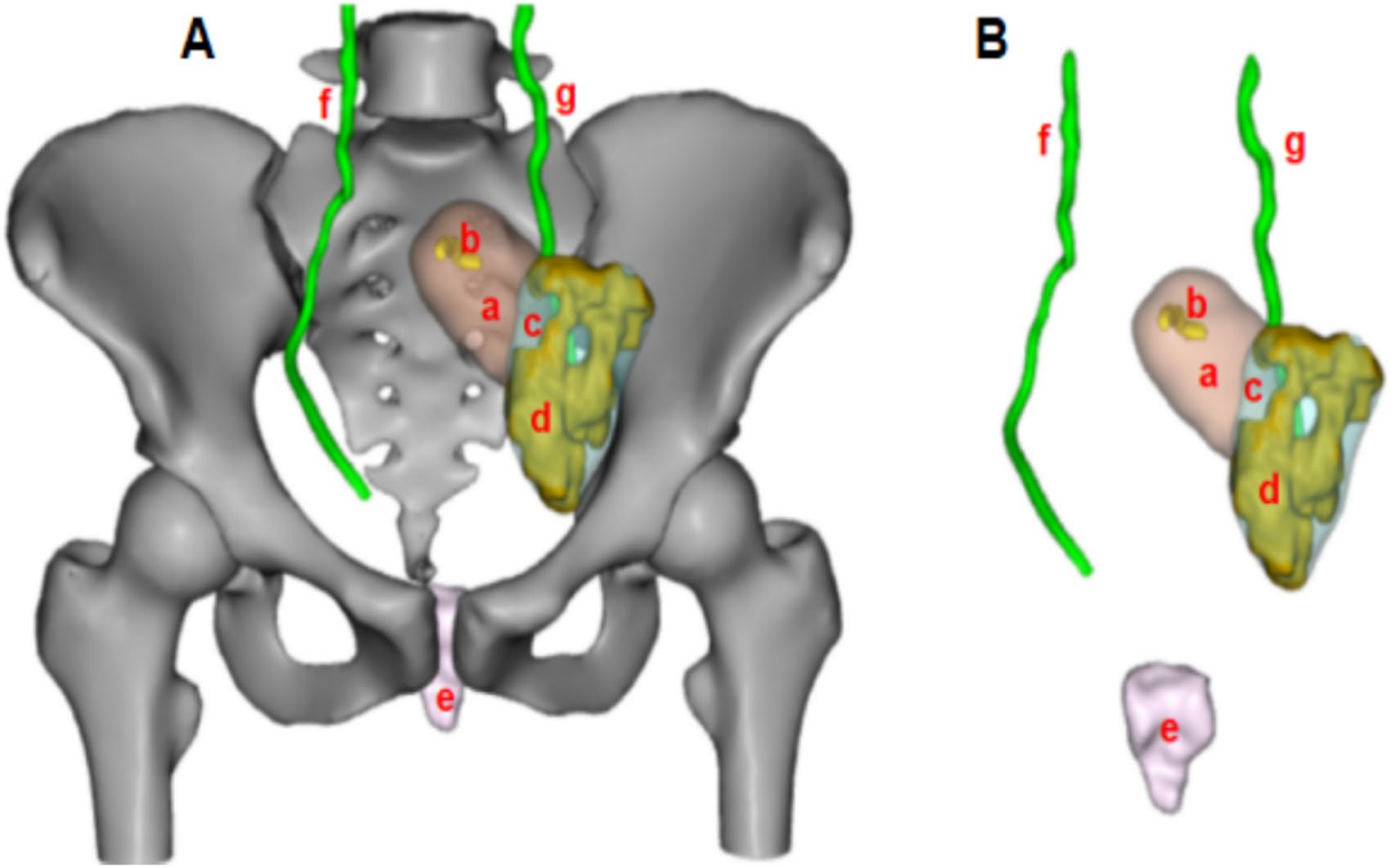
Schematic diagram of the patient’s digital 3D CT reconstruction (Panel **A**: Schematic representation with bony structures; Panel **B**: Schematic representation with bony structures hidden; a: left rudimentary horn uterus; b: uterine cavity; c: left ovary; d: hydrosalpinx of the left fallopian tube; e: vagina; f: right ureter; g: left ureter).

Diagnostic assessment: Based on the gynecological examination and auxiliary investigations, particularly the CT findings, the patient was diagnosed with a complex reproductive tract malformation. To further refine the preoperative diagnosis and guide surgical planning, DICOM images from multiparametric CT scans were processed using medical imaging software for digital 3D CT reconstruction (Figure 3). The reconstruction revealed: Left rudimentary horn uterus with cavity, Hydrosalpinx of the left fallopian tube, and absence of the right ovary, fallopian tube and hemi-uterus.

Preoperative diagnosis: Uterine malformation characterized by: left rudimentary horn uterus, hydrosalpinx of the left fallopian tube and absence of the right ovary, fallopian tube and hemi-uterus.

Surgical planning and communication: The digital 3D CT reconstruction demonstrated that the left rudimentary horn uterus was positioned high, at the level of the anterior superior iliac spine. After thorough discussion with the patient and family, it was determined that if the rudimentary horn uterus could not be mobilized and connected to the vagina during surgery, a resection would be performed. The primary goals were to relieve reproductive tract obstruction and preserve fertility to the greatest extent possible.

**Figure 4:**
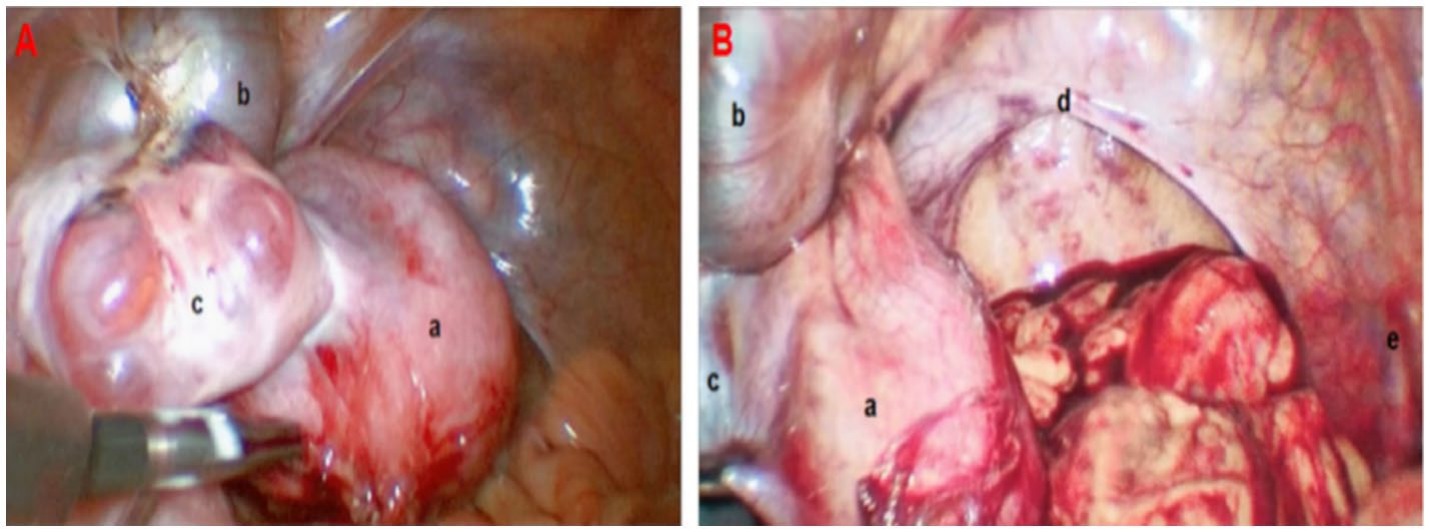
Laparoscopic findings observed in the patient (Panel **A**: Left hemipelvic view; Panel **B**: Comprehensive pelvic view; a: left rudimentary horn uterus; b: hydrosalpinx of the left fallopian tube; c: left ovary; d: bladder peritoneal reflection at the top of the vagina; e: absence of the right ovary and fallopian tube).

Operative findings (April 19, 2024, Laparoscopy): Left pelvic wall: Isolated rudimentary horn-like uterine tissue measuring approximately 5 cm × 4 cm × 3 cm, with a full appearance and rich surface vasculature. Cervix: Absent. Left fallopian tube: Distally dilated and hydropic (4 cm × 3 cm), containing chocolate-like fluid, with closed fimbriae. Left ovary: Multiple small cysts (peanut-sized) on the surface. Right ovary and fallopian tube: Absent. Vagina: Length approximately 8 cm, with a blind-ending vault. Pelvic cavity: Central pelvis empty, with no right hemi-uterus identified. Part of the greater omentum was adherent to the left rudimentary horn uterus (Figure 4).

Surgical procedure: Consistent with the preoperative 3D CT reconstruction, the left rudimentary horn uterus was confirmed to be high in position, with its blood supply derived solely from branches of the left ovarian artery. Due to these anatomical constraints, it was deemed impossible to mobilize the rudimentary horn and connect it to the vagina. Consequently, a laparoscopic resection of the left rudimentary horn uterus and left fallopian tube was performed.

Postoperative diagnosis: Left rudimentary horn uterus, hydrosalpinx of the left fallopian tube and absence of the right ovary, fallopian tube and hemi-uterus.

Follow-up: The patient was followed for 9 months postoperatively, during which she reported complete resolution of cyclical lower abdominal pain. She recovered well from the surgery, and the procedure successfully alleviated her obstructive symptoms.

## Discussion and conclusions

Müllerian malformations represent a heterogeneous group of congenital anomalies affecting the female reproductive tract, with an estimated incidence ranging from 4 to 7% in the general population (3). These anomalies exhibit a broad spectrum of clinical presentations, ranging from asymptomatic cases to severe reproductive and obstetric complications, including infertility, recurrent pregnancy loss, and dysmenorrhea (2). The classification of Müllerian duct anomalies (MDAs) is primarily based on anatomical features, with the most widely accepted system being the American Society for Reproductive Medicine (ASRM) classification (formerly known as the American Fertility Society classification). However, this classification system has limitations, focusing mainly on uterine anomalies while inadequately describing cervical and vaginal anomalies. It also lacks clear diagnostic criteria, making it difficult to accurately classify complex anomalies (such as the case presented in this article) (6). To address these issues, the European Society of Human Reproduction and Embryology/European Society of Gynecological Endoscopy (ESHRE/ESGE) proposed a new classification system for Müllerian duct anomalies. This system is based on uterine anomalies and classifies cervical and vaginal anomalies independently and in parallel, with subtypes divided according to the degree of anomaly and clinical significance (from mild to severe). The specific classifications are as follows: uterine anomalies (U0-U6), cervical anomalies (C0-C4), and vaginal anomalies (V0-V4) (3). According to the ESHRE/ESGE classification, the case in this article is categorized as U5aC4V0. It is noteworthy that prior to the ESHRE/ESGE consensus, Oppelt et al. proposed the Vagina Cervix Uterus Adnex-associated Malformation (VCUAM) classification system (7). This system classifies anomalies of the vagina (V), cervix (C), uterus (U), adnexa (A), and associated malformations (M) in a more detailed and component-based manner. While the ESHRE/ESGE system offers a generalized and widely adopted framework, the VCUAM classification remains clinically relevant for describing complex anomalies that involve adnexal structures, a aspect not fully captured by the ESHRE/ESGE system. According to the VCUAM classification, the case in this article is defined as V0C2bU4aA3aM0, representing a rare and complex Müllerian duct anomaly. The combination of a cavitated rudimentary horn with ipsilateral adnexal pathology (hydrosalpinx) and contralateral agenesis of the uterus, fallopian tube, and ovary, as seen in our patient, is exceptionally rare. Review of the literature reveals that unilateral Müllerian and adnexal agenesis is uncommon, and its association with a functional, obstructed rudimentary horn is even more seldom reported (8). Acién and Acién, in their comprehensive review of complex malformations, emphasized the spectrum of associated adnexal anomalies, which can significantly impact clinical presentation and surgical strategy (2). Our case aligns with their observations that such complex anomalies often present with pain and require meticulous preoperative mapping. Similarly, case series focusing on rudimentary horns highlight the risk of obstruction and endometriosis, but cases with concomitant unilateral adnexal absence are infrequently detailed (9).

In this case, the patient presented with primary amenorrhea and cyclic lower abdominal pain, which are hallmark symptoms of Müllerian anomalies (1). The absence of menstruation despite normal secondary sexual characteristics often points to an underlying anatomical abnormality, such as a Müllerian duct anomaly. The diagnostic complexity in this case was further compounded by the presence of a unilateral isolated rudimentary horn uterus with associated anomalies, including the unilateral absence of the ovary, fallopian tube and hemi-uterus. These findings underscore the necessity of comprehensive imaging modalities, such as pelvic CT and digital 3D reconstruction, to accurately delineate the anatomical abnormalities and guide clinical decision-making.

While magnetic resonance imaging (MRI) and three-dimensional (3D) ultrasonography are rightly considered the gold standards for evaluating Müllerian anomalies due to their excellent soft-tissue resolution and lack of ionizing radiation (10), digital 3D CT reconstruction offers unique advantages in specific, complex clinical scenarios. MRI excels in characterizing endometrial and myometrial tissues, while 3D ultrasound provides real-time, dynamic assessment. In the present case, however, contrast-enhanced CT with 3D reconstruction was selected due to its immediate availability during the patient’s acute pain exacerbation and its exceptional capability for high-resolution spatial modeling. This modality provided superior bony anatomical localization and unparalleled clarity in depicting the spatial relationships within a severely distorted pelvis. The digital 3D CT reconstruction proved instrumental in delineating the complex anatomy—specifically the elevated position of the isolated rudimentary horn relative to the pelvic brim and its connection to the adnexa—thereby providing an indispensable “surgical roadmap” for the subsequent laparoscopic procedure. It is crucial to acknowledge the limitations of CT, including radiation exposure and inferior soft-tissue contrast compared to MRI. Therefore, the use of 3D CT should be judiciously reserved for selected cases where other modalities are inconclusive, or when urgent surgical planning necessitates exquisite anatomical detail beyond the scope of ultrasound or MRI.

The association between obstructive Müllerian anomalies and adenomyosis, though uncommon in adolescents, underscores the importance of timely intervention. The postulated mechanism involves outflow obstruction leading to increased intrauterine pressure and retrograde menstruation, which may facilitate myometrial invasion by endometrial tissue (11). This pathophysiological link is supported by reviews discussing the sequelae of obstructive anomalies in young women (12). In the present case, preoperative sonographic findings of heterogeneous myometrial echogenicity raised suspicion for adenomyosis, likely aggravated by the obstructive rudimentary horn. This pathophysiological continuum highlights the necessity of early surgical correction to relieve obstruction and prevent secondary complications such as adenomyosis and potential endometriosis, as discussed in the literature focusing on obstructive Müllerian anomalies (12).

Following laparoscopic resection of the obstructed rudimentary horn and salpinx, the patient experienced complete resolution of cyclic pain, affirming the value of prompt and targeted surgical management. Long-term care for such patients must also incorporate fertility preservation strategies, including conservation of the contralateral ovary (13). As emphasized in the review by Rackow and Arici on reproductive performance in Müllerian anomalies, the preservation of functional ovarian tissue is paramount for future fertility potential, particularly in complex cases like unilateral anomalies (14). Patients with such conditions often require early and ongoing counseling regarding their reproductive options and the possible need for assisted reproductive technologies. Multidisciplinary collaboration, involving gynecologists, radiologists, and reproductive endocrinologists, is often necessary to address the diverse needs of these patients, an approach strongly advocated for in the management of complex Müllerian anomalies (2).

In conclusion, this case illustrates the diagnostic and therapeutic challenges associated with rare Müllerian malformations. The use of advanced imaging techniques, such as digital 3D CT reconstruction, plays a crucial role in optimizing patient outcomes. Further research is needed to refine existing classification systems, improve diagnostic accuracy, and enhance our understanding of the underlying pathophysiology of these complex anomalies.

## Data Availability

The original contributions presented in the study are included in the article/supplementary material, further inquiries can be directed to the corresponding author.
